# Methotrexate and Cetuximab—Biological Impact on Non-Tumorigenic Models: In Vitro and In Ovo Assessments

**DOI:** 10.3390/medicina58020167

**Published:** 2022-01-22

**Authors:** Andreea M. Kis, Ioana Macasoi, Corina Paul, Matilda Radulescu, Roxana Buzatu, Claudia G. Watz, Adelina Cheveresan, Delia Berceanu, Iulia Pinzaru, Stefania Dinu, Aniko Manea, Marioara Poenaru, Claudia Borza, Cristina A. Dehelean

**Affiliations:** 1 Department of ENT, “Victor Babeş” University of Medicine and Pharmacy Timisoara, Eftimie Murgu Square No. 2, 300041 Timișoara, Romania; kis.andreea@umft.ro (A.M.K.); marioara.poenaru@gmail.com (M.P.); 2 Departament of Toxicology and Drug Industry, Faculty of Pharmacy, “Victor Babeş” University of Medicine and Pharmacy Timisoara, Eftimie Murgu Square No. 2, 300041 Timişoara, Romania; macasoi.ioana@umft.ro (I.M.); iuliapinzaru@umft.ro (I.P.); cadehelean@umft.ro (C.A.D.); 3 Research Center for Pharmaco-Toxicological Evaluations, Faculty of Pharmacy, “Victor Babes” University of Medicine and Pharmacy Timisoara, Eftimie Murgu Square No. 2, 300041 Timisoara, Romania; farcas.claudia@umft.ro; 4 Department of Pediatrics, “Victor Babeş” University of Medicine and Pharmacy, Eftimie Murgu Square No. 2, 300041 Timișoara, Romania; paul.corina@umft.ro; 5 Department of Microbiology, Faculty of Medicine, “Victor Babes” University of Medicine and Pharmacy Timisoara, Eftimie Murgu Square No. 2, 300041 Timisoara, Romania; berceanu.delia@umft.ro; 6 Department of Dental Aesthetics, Faculty of Dental Medicine, “Victor Babeş” University of Medicine and Pharmacy Timisoara, 9 No., Revolutiei Bv., 300041 Timişoara, Romania; 7 Departament of Pharmaceutical Physics, Faculty of Pharmacy, “Victor Babeș” University of Medicine and Pharmacy Timisoara, Eftimie Murgu Square No. 2, 300041 Timișoara, Romania; 8 Department of Pharmacology, Faculty of Medicine, “Victor Babes” University of Medicine and Pharmacy Timisoara, Eftimie Murgu Square No. 2, 300041 Timisoara, Romania; cheveresan.adelina@umft.ro; 9 Department of Pedodontics, Faculty of Dental Medicine, “Victor Babeş” University of Medicine and Pharmacy Timisoara, 9 No., Revolutiei Bv., 300041 Timişoara, Romania; dinu.stefania@umft.ro; 10 Pediatric Dentistry Research Center, Faculty of Dental Medicine, “Victor Babeş” University of Medicine and Pharmacy Timisoara, 9 No., Revolutiei Bv., 300041 Timişoara, Romania; 11 Department of Neonatology, Faculty of Medicine, “Victor Babes” University of Medicine and Pharmacy Timisoara, Eftimie Murgu Square No. 2, 300041 Timisoara, Romania; aniko180798@yahoo.com; 12 Department of Pathophysiology, Faculty of Medicine, “Victor Babes” University of Medicine and Pharmacy Timisoara, Eftimie Murgu Square No. 2, 300041 Timisoara, Romania; borza.claudia@umft.ro

**Keywords:** methotrexate, cetuximab, HaCaT, cell viability, HET-CAM assay, RT-PCR

## Abstract

*Background Objectives:* The neoplastic process remains a major health problem facing humanity. Although there are currently different therapeutic options, they raise a multitude of shortcomings related to the toxic effects associated with their administration. Methotrexate (Met) and Cetuximab (Cet) are two basic chemotherapeutics used in cancer practice, but notwithstanding despite many years of use, the mechanisms by which the multitude of side-effects occur are not yet fully understood. Thus, the present study focused on the in vitro and in ovo evaluation of the associated toxic mechanisms on keratinocytes, keys cells in the wound healing process. *Materials and Methods:* The two chemotherapeutics were tested in eight different concentrations to evaluate keratinocytes viability, the anti-migratory effect, and the influence on the expression of markers involved in the production of cell apoptosis. In addition, the potential irritating effect on the vascular plexus were highlighted by applying the in ovo method, chick chorioallantoic membrane (HET-CAM). *Results*: The results revealed that Met induced decreased cell viability as well as increased expression of pro-apoptotic genes. In the vascular plexus of the chorioallantoic membrane, Met caused vascular irritation accompanied by capillary hemorrhage and vascular stasis. *Conclusions*: Summarizing, Cet presents a safer toxicological profile, compared to Met, based on the results obtained from both in vitro (cell viability, wound healing, RT-PCR assays), and in ovo (HET-CAM assay) techniques.

## 1. Introduction

Worldwide, over 500,000 new cases of head and neck squamous cell carcinoma (HNSCC) are reported annually [1]. Rich desmoplastic reaction is considered a pathognomonic feature of HNSCC, further driving the hypothesis that the tumor microenvironment is presumably a key component in pathophysiology of this cancer [2]. However, the clinicopathologic response of HNSCC to conventional protocols, including surgical excision and chemo-radiotherapy, suggests that treatment strategies directed toward tumor cells alone are inadequate and targeting non-cancerous cells in the tumor microenvironment may improve clinical outcomes. The importance of the tumor microenvironment and its crosstalk with cancer cells are increasingly being recognized as important steps in the pathogenesis and progression of several cancers [2]. In other words, revealing the micro-environment and behavior of non-tumoral cells under specific conditions could be considered a first step towards a complex understanding of the tumoral cell micro-environment needs. Regarding chemo-therapy protocols, drug options for single-agent therapy of HNSCC include cisplatin, carboplatin, paclitaxel, docetaxel, 5-fluorouracil, methotrexate, cetuximab, afatinib, or capecitabine [3]. A suitable drug option in advanced cases is Methotrexate (Met)—a folic acid antagonist which acts as a ligand for the folate receptor, that has been associated with abnormally rapid cell growth and exhibits anti-inflammatory and immunosuppressant activities, being used for long-term treatment schemes in leukemia and rheumatic diseases and advanced cancer head and neck treatment [4]. However, several toxic effects have been reported, such as acute mucosal ulcers, bone marrow suppression, skin erosions, and skin toxicity with keratinocyte dystrophy and alopecia [3,5,6]. Various new formulations have been studied in order to reduce the toxicity associated with Methotrexate administration. This formulation is based on new drug delivery systems, such as liposomes or nanoparticles [7]. The combination of chemotherapy and radiation therapy, called chemoradiation, is currently one of the most innovative approaches in cancer treatment. For this reason, nanoparticles have received special attention because they can serve as a delivery system for chemotherapeutic substances [8]. One such example is the study by Faghfoori et al. [9], in which they synthesized albumin coated bismuth sulfide nanoparticles and conjugated them with methotrexate. They showed that this combination causes a decrease in cell viability, and, in addition, the nanoparticles have the ability to be radiosensitive, which leads to increased therapeutic effect [9]. A similar study was conducted by Nosrati and co-workers, through which they conjugated methotrexate with Bi_2_S_3_–Au nanoparticles. After 20 days of administration, using a murine model, they noticed that the tumor had been completely eradicated [10]. In addition to toxic reactions, antitumor therapy also faces the problem of developing resistance to treatment of tumor cells. A major role in resistance to therapy is played by cancer stem cells. In a study conducted to obtain a targeted effect on cancer stem cells, a therapeutic strategy of 22-bp double-stranded STAT3 decoy ODNs treatment with methotrexate was developed. Following in vitro testing of triple-negative MDA-MB-231 breast cancer cells, this innovative therapy has been shown to be effective and has promising potential for future antitumor therapy [11].

Another cytostatic used in therapy is Cetuximab (Cet)—a human–mouse chimeric anti-epidermal grow factor receptor (EGFR) containing the human IgG1 constant region that binds specifically to the external domain of EGFR and blocks ligand binding and receptor activation [12]. Therefore, it is activated in many types of epithelial cancers, including head and neck, esophageal, lung, liver, pancreatic, colon, skin, and bladder. EGFR is over-activated by mutation, amplification, or overexpression. In addition, high levels of EGFR in tumors and metastases is correlated with poorer patient outcome [13]. Nevertheless, the administration of Cet is endorsed when EGFR levels are high, but there are also adverse effects induced by Cet treatment, reactions that are usually related to skin toxicity, such as infusion reaction, corneal erosion, and keratitis [14]. It is believed that the adverse reactions are provoked by the glycan contents on the Fab regions; therefore, a recent study [15] developed an humanized anti-EGFR monoclonal antibody based on cetuximab by humanizing the Fab regions of Cet leading to a minimum content of glycan. The novel monoclonal antibody showed promising results on different tumor cell lines such as A431, FaDu, and NCI-H292; furthermore, the compound is currently under clinical development (phase 1/2).

Another strategy to avoid the skin side-effects induced by EGFR inhibitors, such as Cet, was recently approached by Lacouture et al. [16] in a phase I clinical trial, by proposing a topical therapy with a BRAF inhibitor. The results showed that this treatment induced an improvement of the acneiform rash of the patients that presented a grade two rash.

**Figure 1:**
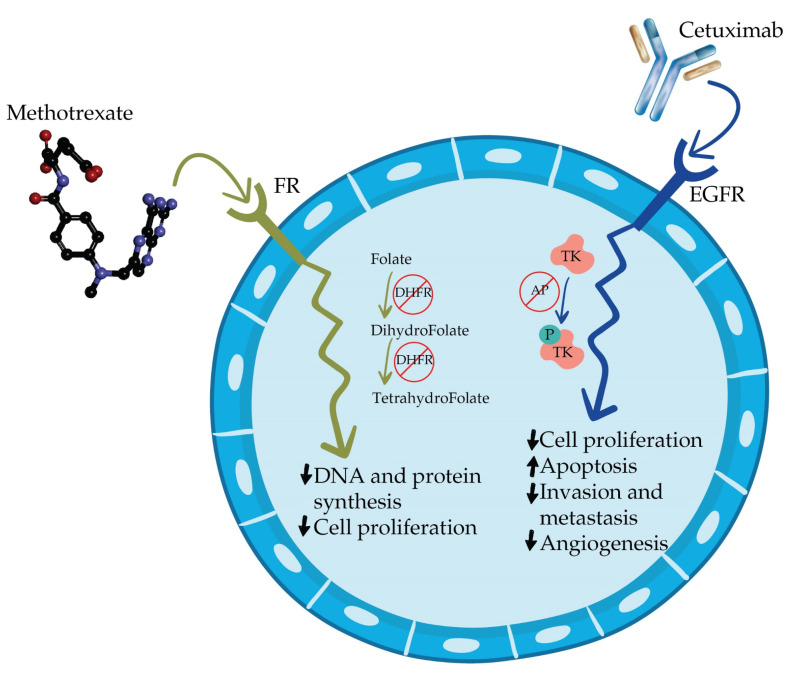
Mechanism of antitumor action of Methotrexate and Cetuximab. Methotrexate is an antagonist of the folate receptor (FR) causing the blockade of the enzyme dihydrofolate reductase (DHFR) having consequently inhibition of protein and DNA synthesis and cell proliferation. Cetuximab acts on the EGFR receptor by inhibiting the autophosphorylation (AP) of tyrosine kinase (TK), thereby terminating the inhibition of cell proliferation, invasion, metastasis, and angiogenesis, while stimulating the process of apoptosis.

Figure 1 schematically shows the antitumor mechanisms of action of Methotrexate and Cetuximab.

Since adverse reactions are closely related to skin toxicity, evaluation of the two cytostatics (Methotrexate and Cetuximab) on non-tumorigenic skin cells may provide essential details in understanding the secondary effects regarding the cytotoxic effect of Cetuximab and Methotrexate. In this regard, immortalized human keratinocyte cultures were employed as an in vitro model, because they are considered keys cells in the wound healing process and also show a low expression of folate receptors. Under homeostatic conditions, keratinocytes differentiate and mature from proliferating nucleated basal cells to the highly differentiated, nucleus-free corneocytes. Each stage of differentiation is characterized by the expression of structural proteins, such as keratins (K) and lipids [17]. Resting keratinocytes produce epidermal growth factor receptor (EGFR) ligands and vascular endothelial growth factor (VEGF), however when activated by bacterial products or by direct damage of UV light or chemicals, the expression of cytokines and chemokines changes. Nevertheless, cultured human keratinocytes are frequently employed for studies of various functions encountered in chronic inflammatory skin diseases [18].

The main aim of the study was to evaluate in vitro and in ovo the effects of methotrexate and cetuximab to determine their toxic potential in healthy cells. Thereby, the present study focuses on the evaluation of different concentrations (5, 15, 20, 30, 60, 90, 120, 150 µg/mL) of Methotrexate and Cetuximab on HaCaT cell line, in terms of cell viability and anti-proliferative/anti-migratory capacity, when a stimulation time interval of 24 h was employed. To evaluate the mechanism associated with cell death, it was decided to apply the RT–PCR method by which the effect of Methotrexate and Cetuximab was evaluated in concentration of 150 µg/mL in the expression of pro-apoptotic genes (Bax and Bad) and anti-apoptotic (Bcl-xL and Bcl-2). In addition, the potential irritant effect of the two cytostatics on the vascular plexus was also examined in ovo, by employing the chick chorioallantoic membrane (HET-CAM) assay.

## 2. Materials and Methods

### 2.1. Cell Line and Cell Culture Conditions

Human immortalized keratinocytes—HaCaT (300493; CLS Cell Lines Service GmbH) cells were used in the current study to assess the biological impact of two different cytostatics (Methotrexate and Cetuximab). The culture conditions for HaCaT cell line consists of: Dulbecco’s Modified Eagle’s Medium (DMEM 30-2002™) high glucose (4.5 g/L) media, with 15 mM HEPES (cat. no. H3375), and 2 mM L-glutamine (cat. no. G7513 supplemented with 10% Fetal Calf Serum (cat. no. 12103C) and 1% antibiotic mixture of 100 U/mL penicillin: 100 µg/mL streptomycin (cat. no. P4333), to avoid a possible bacterial infection provided by Sigma-Aldrich, Munich, Germany. The cell cultures were maintained under standard conditions, as described before [19] by providing an humidified atmosphere enriched in 5% CO2, at 37 °C by means of the Steri-Cycle i160 incubator (Thermo Fisher Scientific, Inc., Waltham, MA, USA). In addition, all the in vitro experiments were performed under sterile conditions by using a biosafety cabinet, MSC Advantage 12 model (Thermo Fisher Scientific, Inc., Waltham, MA, USA).

### 2.2. Cell Viability Evaluation by Means of Alamar Blue Colorimetric Assay

The effect induced the by test sample (Methotrexate and Cetuximab) on the human immortalized keratinocytes (HaCaT) viability was evaluated by performing the Alamar Blue colorimetric test, as previously described [20,21]. In brief, 1 × 10^4^ cells per well were seeded onto 96-well culture plates and incubated until a confluence of 70–80% was reached. Afterwards, the old medium was removed, and the cells were treated with cell culture medium containing different concentrations (5, 15, 25, 30, 60, 90, 120, 150 µg/mL) of test samples—Met and Cet. The control cells were maintained under the same conditions as the test-treated ones; however, they were exposed only to culture medium. To quantify the cell viability percentage, the absorbance of each well (control and test-treated) was determined spectrophotometrically at two wavelengths (570 and 600 nm) by means of the microplate reader (xMarkTM Microplate, Bio-Rad Laboratories, Hercules, CA, USA) and the formula applied to determine the cell viability percentage is presented in our previous studies [22].

### 2.3. Scratch Assay Technique—A Wound Healing Method

To evaluate the antiproliferative impact induced by different concentrations (5, 15, 20, 30, 60 µg/mL) of Methotrexate and Cetuximab on human immortalized keratinocytes behavior, the scratch assay method was employed. This is a facile and economic assay that provides meaningful data regarding the antimigratory effect induced by test samples on both tumorigenic and normal cell cultures [23]. The principle of this technique consists in removing a small part of the cell monolayer by making a scratch along the well diameter, thus obtaining a cell-free surface in each well [24]. This area is constantly supervised to observe the migratory and proliferative capacity of the cells (control versus test-treated cells). An initial density of 2 × 10^5^ HaCaT cells/well were seeded onto a 12-well plate. The cells were allowed to attach to the bottom of the plate and were incubated until the cell monolayer reached an approximately confluence of 80%. The next step consisted of removing the old medium and obtaining the cell-free area by making a scratch in the middle of the well using a sterile pipette tip. Afterwards, the scratched monolayer and all the cellular debris were removed by washing each well with 1x PBS. After the washing step, the cells were stimulated with five different concentrations (5, 15, 20, 30, 60 µg/mL) of Methotrexate and Cetuximab or treated only with culture media (control cells) and the cell migration was supervised by making pictures at different intervals using an inverted microscope (Olympus IX73, Tokyo, Japan) documented with an integrated DP74 camera (Olympus, Tokyo, Japan). The scratched widths were determined using the scale measurement function of the camera software (CellSense Dimension). To calculate the migration rate, the following formula was applied [24]:

$$Scratch\;closure\;after\;24\;h\;(\%)\;=\;\frac{Scratch\;surface\;(0\;h)\;-\;Scratch\;surface\;(24\;h)}{Scratch\;surface\;(0\;h)}\;\times \;100\;\%$$

### 2.4. RT-PCR Analysis

A RT-PCR analysis was conducted in order to establish the influence of Met and Cet on gene expression. To determine the effects of the samples on the Bad, Bax, Bcl-2, and Bcl-xL genes, human keratinocytes were cultured in 6-well plates at 10^6^ cells/well. After reaching a 90% confluence, the cells were stimulated for a period of 24 h with Met and Cet in a concentration of 150 µg/mL. Total RNA was isolated from HaCaT cells using Trizol (cat. no. 15596026) purchased from Thermo Fisher Scientific, Inc. (Waltham, MA, USA) and Quick-RNA ™ (cat. no. R1054) purification kit (Zymo Research) was used for RNA purification. The next step of the procedure was to obtain cDNA by reverse transcription reaction using Maxima^®^ First Strand cDNA Synthesis Kit (Fermentas, cat no. K1641). Quantitative real-time PCR analysis was performed using the Quant Studio 5 real-time PCR system (Thermo Fisher Scientific, Inc., Waltham, MA, USA). The analyzed mixture consisted of 20 µL solution and contained the following: i) Power SYBR-Green PCR Master Mix (Thermo Fisher Scientific, Inc., Waltham, MA, USA, cat. no. 4309155), cDNA samples, the sense and antisense primer and pure water. The primers used are shown in Table 1 and included: 18S (housekeeping genes), Bax, Bcl-2 (Thermo Fisher Scientific, Inc., Waltham, MA, USA), Bad (Eurogentec, Seraing, Belgium), and Bcl-xL (Eurogentec, Seraing, Belgium).

### 2.5. HET–CAM Method

The HET-CAM method was applied to determine the possible irritant effect of Methotrexate and Cetuximab. In order to perform this test, the following stages of egg preparation were performed: (i) the eggs were washed and disinfected with alcohol of 70% (*v*/*v*) concentration; (ii) the eggs were dated and placed in a horizontal position in the incubator at a constant temperature of 37 °C; (iii) on the 4th day of incubation, the eggshell was carefully perforated and a volume of approximately 7 mL of albumen was extracted to allow the chorioallantoic membrane to detach from the upper part of the eggshell, after which the perforation was covered with adhesive tape, and the eggs were reintroduced into the incubator; and (iv) on the 5th day of incubation, at the upper level of the egg a window was cut to allow the visualization of the chorioallantoic membrane vessels, the perforation was covered with adhesive tape, and eggs were reintroduced into the incubator until the day the experiment began. The HET-CAM test was performed on the 10th day of incubation. A positive control of 1% sodium dodecyl sulfate (SDS) and a negative control of distilled water were used to verify and quantify the irritating effect of the samples. Methotrexate and Cetuximab were tested at the highest concentrations used in the viability test (150 µg/mL). Thus, at the level of the chorioallatoic membrane a volume of 500 µL of positive control, negative control, respectively, of samples was applied. The changes in the vascular plexus followed and described were hemorrhage, lysis, and vascular coagulation. These irritant effects were observed for a period of 5 min. Quantification of the irritant effect was performed by photographing the membrane before applying the samples (T0) and 5 min after applying them (T5). Photographs and microscopic evaluation of sample-induced effects were performed using Axio CAM 105 color, Zeiss, Discovery 8 Stereomiscroscope and Image J software v 1.50e software (U.S. National Institutes of Health, Bethesda, MD, USA). To quantify the vascular effects, the analytical method for calculating the irritation score (IS) was used with the formula described above [25,26]:

$$\mathrm{IS}=5\times \frac{301-H}{300}+7\times \frac{301-L}{300}+9\times \frac{301-C}{300}$$

Irritation score is a parameter used to quantitatively determine the toxic and irritating effect of a compound by measuring the time at which vascular changes occur such as hemorrhage (H), vascular lysis (L) and coagulation (C). Depending on the value of the irritation score, the substances can be classified as follows: (i) non-irritating (IS = 0–0.9); (ii) irritating (IS = 1–8.9), and (iii) severe irritating (IS = 9–21) [27].

### 2.6. Statistical Analysis

GraphPad Prism version 5 and GraphPad Prism version 8.3.0 software (GraphPad Software, San Diego, CA USA) were used to analyze and present the statistical analysis data. One-way ANOVA test was performed to determine the statistical differences of test samples (Methotrexate and Cetuximab) versus control, followed by Tukey’s post-test in the case of cell viability and migration (* *p* < 0.05, ** *p* < 0.01, *** *p* < 0.001) and Dunnett’s post-test in the case of gene expression (* *p* < 0.05, ** *p* < 0.01 and **** *p* < 0.00001).

## 3. Results

### 3.1. Cell Viability Assessment

**Figure 2:**
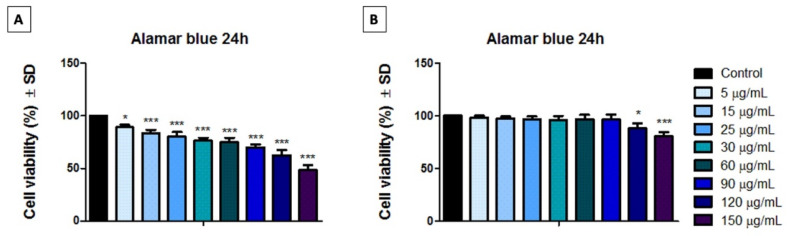
(**A**) Cell viability rate of human immortalized keratinocytes (HaCaT) after stimulation with different concentrations (5, 15, 25, 30, 60, 90, 120, 150 µg/mL) of Methotrexate for 24 h. (**B**) Cell viability rate of human immortalized keratinocytes (HaCaT) after stimulation with different concentrations (5, 15, 25, 30, 60, 90, 120, 150 µg/mL) of Cetuximab for 24 h. The graph bars are expressed as cell viability percentage (%) normalized to control cells (cells treated with culture medium). One-way analysis of variance (ANOVA) test was performed to determine the statistical differences of test sample versus control, followed by Tukey’s multiple comparisons post-test (* *p* < 0.05, *** *p* < 0.001).

To evaluate the effect induced by both cytostatics (Methotrexate and Cetuximab) on human immortalized keratinocytes (HaCaT), the Alamar blue colorimetric test was performed by employing a stimulation time of 24 h. The results are depicted in Figure 2, as follows: Figure 2A presents the cell viability rate induced by Methotrexate, while Figure 2B shows the viability rate of HaCaT cells treated with Cetuximab.

As presented in Figure 2, the results indicate that the human immortalized keratinocytes (HaCaT) population was more affected after exposure to different concentrations of Methotrexate for 24 h (Figure 2A), compared to the results obtained when the cells were treated with Cetuximab under the same treatment conditions (Figure 2B). Cetuximab did not induce a significant cell viability decrease of human immortalized keratinocytes when the concentrations applied ranged between 5 to 90 µg/mL, after a stimulation time of 24 h; under these conditions, the cell viability rates were above 96%. However, when the human immortalized keratinocytes were treated with the same concentrations (5, 15, 25, 30, 60, 90 µg/mL) of Methotrexate, the HaCaT cells manifested a dose-dependent viability decrease, as follows: 89%, 83.2%, 80%, 76.4%, 74.89%, and 70.02%, respectively.

Nevertheless, when high concentrations (120 and 150 µg/mL) of both cytostatics (Methotrexate and Cetuximab) were used, a statistically significant reduction of HaCaT viable population was recorded, as follows: (i) Met led to a viability of 62.48% and 48.55%, respectively; (ii) keratinocyte monolayers treated with Cet showed a lower cytotoxic activity, however still statistically significative by led to a cell viability of 88.2% and 80.4%, respectively.

### 3.2. Evaluation of Antiproliferative and Antimigratory Capacity

**Figure 3:**
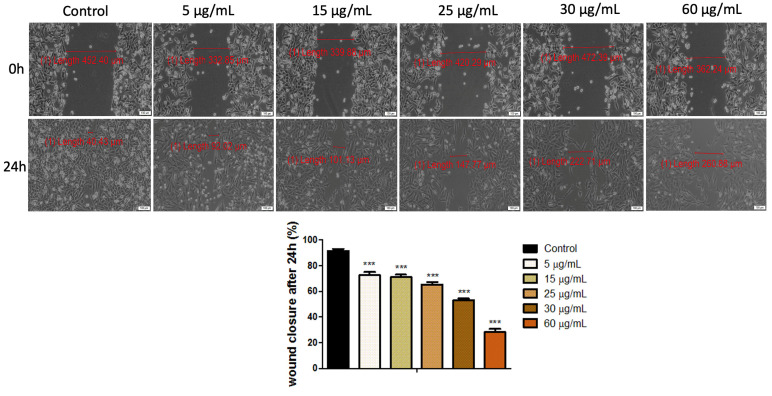
Wound healing rate of human immortalized keratinocytes (HaCaT) after exposure to different concentrations (5, 15, 20, 30, 60 µg/mL) of Methotrexate. The migratory capacity of HaCaT was monitored by taking pictures of the scratched area initially and 24 h post stimulation. Graph bars are calculated as the percentage of the scratched surface at 24 h, compared to the initial scratched surface (0 h). Scale bars denote 100 μm. The results represent the mean values ± standard deviation (SD) of three independent experiments. One-way ANOVA was employed to determine the statistical differences, followed by a Tukey post-test (*** *p* < 0.001).

**Figure 4:**
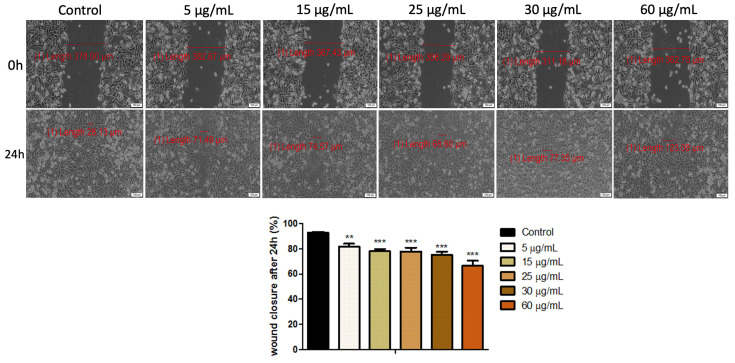
Wound healing rate of human immortalized keratinocytes (HaCaT) after exposure to different concentrations (5, 15, 20, 30, 60 µg/mL) of Cetuximab. The migratory capacity of HaCaT was monitored by taking pictures of the scratched area initially and 24 h post stimulation. Graph bars are calculated as the percentage of the scratched surface at 24 h, compared to the initial scratched surface (0 h). Scale bars denote 100 μm. The results represent the mean values ± standard deviation (SD) of three independent experiments. One-way ANOVA was employed to determine the statistical differences, followed by a Tukey post-test (** *p* < 0.01; *** *p* < 0.001).

Keratinocytes are the executors of the re-epithelialization process due to their migratory and proliferative features. Regarding this aspect, the evaluation of the antiproliferative and antimigratory profile of HaCaT cells after stimulation with both cytostatics (Methotrexate and Cetuximab) represents an important aspect. The assessment was realized by performing the wound healing method and the results obtained are presented in Figure 3—for Methotrexate and Figure 4—for Cetuximab.

Figure 3 shows that the migratory and proliferative capacity of human immortalized keratinocytes (HaCaT) was significantly inhibited after treatment with several concentrations of Methotrexate (5, 15, 20, 30, 60 µg/mL), when compared to control cells—the cells treated with specific culture medium. The scratch closure rate determined at 24 h post-treatment respected a dose-dependent pattern; the cells showed a wound healing percentage of 72.54% when exposed to the lowest concentration (5 µg/mL) of methotrexate, reaching to a wound closure rate of only 28.31% when stimulated with the concentration of 60 µg/mL.

Figure 4 presents the migratory and proliferative potential of human immortalized keratinocytes (HaCaT) treated with five different concentrations (5, 15, 20, 30, 60 µg/mL) of Cetuximab. It can be easily observed that Cetuximab induced a moderate inhibitory capacity on the migration and proliferation behavior of HaCaT monolayer in a dose-dependent manner, the cells showing wound closure percentages of 81.95% after treatment with 5 µg/mL of Cetuximab; the rate decreasing up to 66.51% when the cells were exposed to 60 µg/mL of Cetuximab.

### 3.3. Gene Expression

**Figure 5:**
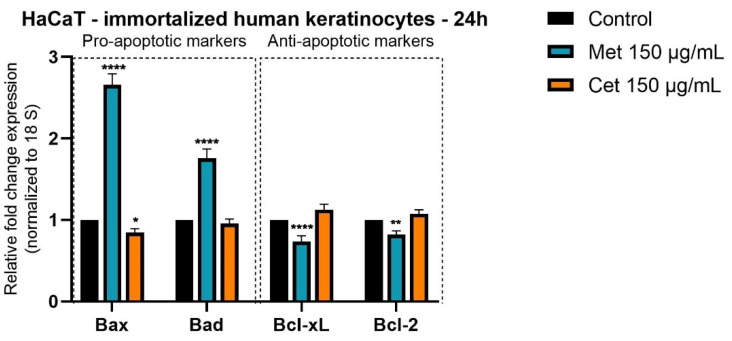
Relative fold expression of mRNA expression of pro- and anti-apoptotic genes in HaCaT cells after stimulation with Methotrexate and Cetuximab (150 µg/mL) for 24 h. Data represent the mean values ± SD of three independent experiments. One-way ANOVA with Dunnett’s post-test was used for statistical analysis versus unstimulated control cells (* *p* < 0.05,** *p* < 0.01 and **** *p* < 0.00001).

Since there were effects of decreased cell viability in the case of Methotrexate at a concentration of 150 µg/mL, and Cetuximab has a weak effect on cell viability at the same concentration, we further evaluated the effect of the two chemotherapeutics on gene expression involved in apoptosis. Two pro-apoptotic genes (Bax and Bad) and two anti-apoptotic genes (Bcl-xL and Bcl-2) were chosen. The dose of 150 µg/mL of Methotrexate causes a significant increase of the pro-apoptotic markers, while on the anti-apoptotic markers, no obvious changes were registered. At the same time, Cetuximab did not cause significant changes in mRNA expression for any marker studied (Figure 5).

### 3.4. HET-CAM Test

**Figure 6:**
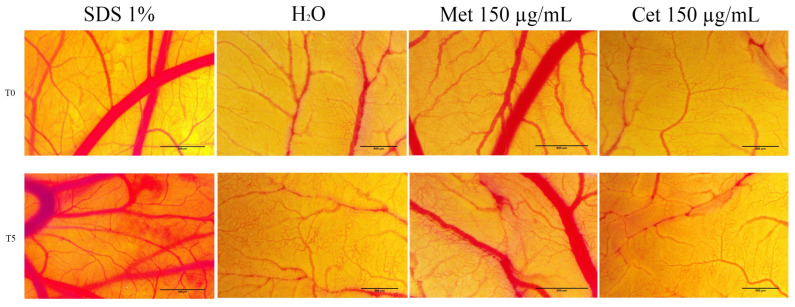
Stereomicroscope images of the CAMs treated with controls (H_2_O used as negative control and sodium dodecyl sulfate—SDS used as positive control) and test samples (Methotrexate and Cetuximab) before inoculation (T0) and five minutes (T5) post-treatment. Scale bars represent 500 µm.

The potential irritant effect of the vascular plexus of Methotrexate and Cetuximab was analyzed using the HET-CAM method. This method has the advantage that it offers a simple method of evaluating the biocompatibility of a compound, based on the calculation of the irradiation score. For a better evaluation of the toxicological profile, a negative control and a positive control were used. The values of the irritation score are presented in Table 2. It can be seen that the highest value of the irritation score was recorded in the case of the positive control, SDS 1%, with a value of 19.41. On the other hand, the lowest value of the irritation score was obtained in the case of the negative control represented by distilled water, of 0.14. Regarding the safety profile of the compounds studied, they have an average irritation score. In the case of Methotrexate, the irritation score calculated 5 min after the application of the sample was 7.83, and in the case of Cetuximab, the irritation score was 1.75. These values are in the range 1–8.9, which indicates that both compounds have a medium irritating effect. However, the irritant effect of Methotrexate is obviously more pronounced than that of Cetuximab. In Figure 6, the changes in the vascular plexus can be seen 5 min after the application of the samples. In the case of SDS 1%, numerous areas with extensive hemorrhages are observed, as well as lysis and strong vascular coagulation. Regarding the negative control, distilled water, at the level of the chorioallantoic membrane, no changes were registered. The concentration of 150 µg/mL of Methotrexate determines the appearance of microhemorrhages and vascular lysis, while Cetuximab at the same concentration determines the appearance of intravascular coagulation and a slight vascular lysis (Figure 6).

## 4. Discussion

The vast majority of cancer patients who are treated with cytostatics agents are prone to develop several forms of cytotoxicity during the active treatment phase. Methotrexate and Cetuximab are part of the treatment options of head and neck squamous cell carcinoma. However, the literature is scarce of data regarding the impact of these two cytostatics on the surrounding non-tumoral tissue, such as human keratinocytes. In this regard, the present paper focuses on revealing insightful details related to: (i) the cell viability of human immortalized keratinocytes treated with different concentrations (5, 15, 25, 30, 60, 90, 120, 150 µg/mL) of both cytostatics (Methotrexate and Cetuximab) for 24 h; (ii) the antimigratory and antiproliferative impact of five different concentrations (5, 15, 20, 30, 60 µg/mL) of both cytostatics (Methotrexate and Cetuximab); (iii) the effects of cytostatics at the highest concentration tested in vitro (150 µg/mL) in the main pro-apoptotic genes (Bax and Bad) as well as in the main anti-apoptotic genes (Bcl-2 and Bcl-xL); and (iv) the potential irritating effect on the vascular plexus of the chorioallantoic membrane.

The results obtained in the current study revealed that human immortalized keratinocytes viability was more affected after exposure to Methotrexate, when compared to the impact induced by Cetuximab, under the same experimental parameters (concentrations and stimulation time interval of 24 h). As presented in Figure 2, HaCaT cells treated with 150 µg/mL of Methotrexate presented a viability rate of 48.55%, while the cells exposed also to the highest concentration (150 µg/mL) of Cetuximab manifested a viability percentage of 80.4%.

Nevertheless, it is also important to highlight the fact that the therapeutic index IC_50_ is very different for the two cytostatics (Met and Cet), as follows: IC_50_ value of Cet is 2000 µg/mL for A549 cell line, 1800 µg/mL for P-H1299 cell line, and 1000 µg/mL for A431 cells [28], while the concentration of Met able to induced half-maximal cell viability (IC_50_) is more reduced (10 µg/mL) for both HeLa and MCF7 cell lines [29].

Based on the cell viability data, the expression of the main genes involved in the apoptosis process was analyzed. As seen in Figure 5, Methotrexate elicited an up-regulation of mRNA expression for the pro-apoptotic markers (Bax and Bad), while Cetuximab did not induce significant changes in the expression of these genes. Finally, by using the in ovo method, it was determined that Methotrexate has a stronger irritant effect compared to Cetuximab. Thus, Figure 6 shows toxic reactions such as haemorrhage following treatment with Methotrexate, while Cetuximab does not cause obvious irritant effects.

In order to emphasize the migratory and proliferative capacity of HaCaT cells by means of wound healing method, the two cytostatics (Methotrexate and Cetuximab) were used at the concentrations that did not induce a reduction of the cell viability by more than 30%. Thus, based on the cell viability percentage observed after applying the Alamar blue assay, only the following concentrations (5, 15, 25, 30, 60 µg/mL) of both drugs were selected for this method. The wound healing test revealed that the proliferative and migratory capacities of HaCaT cells were drastically inhibited after exposure to Methotrexate, compared to Cetuximab, as depicted in Figure 3 and Figure 4, respectively—HaCaT cells manifested a healing rate of 28.31% after 24 h exposure time to 60 µg/mL, while the wound healing percentage of HaCaT cells treated with 60 µg/mL of Cetuximab was around 66%. Therefore, the results obtained for cell viability assessment are consistent with the ones recorded for the wound healing assessment. The moderate antiproliferative effect induced by Cetuximab on human immortalized keratinocytes may be related to the fact that this cytostatic interferes with EGFR activation. EGFR is an inactive monomer on its own, however it is activated after ligand binding and dimerization. This activates the intracellular tyrosine kinase region of EGFR, resulting in the initiation of a signaling pathway involved in cell differentiation, proliferation, migration, angiogenesis, apoptosis, and metastatic spread [30]. EGFR is essentially expressed in normal proliferating keratinocytes in the basal layer of the epidermis and the outer layers of the hair follicle [31]. Moreover, the EGF pathway is involved in keratinocyte survival and proliferation; thus, inhibiting this pathway blocks proliferation, decreases migration, and induces apoptosis of keratinocytes [32]. In addition, EGFR signaling induces epithelial mesenchymal transition in keratinocytes and an immunosuppressive effect [33]. The high antiproliferative effect induced by Methotrexate on human immortalized cells (HaCaT) is caused by another mechanism, compared to Cetuximab and it could be explained by the cell cycle arrest of the treated cells in the G0/G1 phase, as recently reported by Luna et al. [5]. The keratinocytes are maintained at various stages of differentiation in the epidermis and are organized into basal, spinous, granular, and cornified layers that correspond to specific stages of differentiation. The primary function of keratinocytes is to provide the structural integrity of the epidermis, thereby producing an intact barrier to the outside world [17]. The keratinocytes also represent the first line of defense against pathogens in the skin, and therefore play an important role in the innate immune response [34]. This study focuses particularly on the evaluation of Methotrexate and Cetuximab impact on human keratinocyte cultures, since the literature lacks data evidence regarding the cytotoxic effects that can be generated by these cytostatics (Methotrexate and Cetuximab) on non-tumoral micro-environment. Additionally, insightful information on Methotrexate and Cetuximab activity in the wound remodeling process is presented herein, which could contribute to a comprehensive evaluation of the impact of these drugs in the healing process and cell proliferation of a non-tumoral tissue.

The cellular effects of various chemicals can be manifested by promoting or depressing cell death. These effects can be either protective (for example in carcinogenesis) or harmful (for example in impairing physiological functions). Cell survival in the presence of a chemical depends on several factors such as: (i) proliferative state; (ii) enzyme repair capacity, and (iii) increase the expression of proteins involved in promoting or inhibiting the process of cell death [35]. Methotrexate is one of the cornerstones of chemotherapy. The toxic effects associated with this therapy are numerous and involve damage to multiple organs, including the skin. However, the mechanism of toxicity to the skin and, implicitly, to keratinocytes is not fully known [5]. By applying the RT–PCR method, the main expression of pro-apoptotic and anti-apoptotic genes in human keratinocytes was analyzed. The results obtained revealed that in the case of Methotrexate, there is a stimulation of the expression of the pro-apoptotic genes Bax and Bad, while the expression of anti-apoptotic genes is insignificantly altered. On the other hand, Cetuximab does not cause significant changes in gene expression, which remain approximately constant after stimulation of human keratinocytes with a concentration of 150 µg/mL. Concerning the effect of Methotrexate on the induction of apoptosis at the cellular level, there are numerous studies. Thus, Wang and colleagues determined the role of Methotrexate exposure in apoptosis and pointed out that it increases the expression of genes associated with the apoptosis process, including Casp 3 and Bax [36]. It is known that Met increases cytosolic c levels and decreases cytochrome c mitochondrial levels. Through this mechanism, Met increases the transcription factor p53, which induces the transcriptional increase of cytosolic Bax [37]. Due to the therapeutic role played by Met in psoriasis, the effects on genes involved in keratinocyte apoptosis have been studied. Thereby, it has been shown that the effects associated with the induction of apoptosis in keratinocytes are correlated with decreased Bcl-x gene expression [38]. Studies have suggested that by decreasing the level of Bcl-x, there is an increase in the level of cytochrome c mitochondrial, as well as a stimulation of its release in the cytosol, leading to apoptosis of keratinocytes [38]. On the other hand, Cetuximab showed a weak toxic profile in human keratinocytes, the cell viability not being significantly decreased after treatment with it. Jost et al., reported that anti-apoptotic gene, Bcl-xL, depended on EGFR for keratinocyte survival in culture, but this dependence relationship has not been reported for the Bcl-2 gene [39]. In addition, the level of proapoptotic Bcl family members from keratinocyte (Bad, Bak and Bax) is not correlated with EGFR activation [40]. Data from literature suggests that Cet has affinity for major EGFR mutations, such as T790, which are found in many tumor cells. Through this property and since Cet has a lower affinity for normal EGFR in keratinocytes, in the present study, the viability of keratinocytes and the values of markers involved in the apoptosis process were not significantly altered [41]. Based on the directive given by the European Parliament and adopted by the member countries of the European Union, regarding the protection of animals used in scientific research, new research directions have been investigated and introduced [42]. Such a new approach is represented by the use of fertilized chicken eggs, the chorioallatoic membrane allowing the evaluation of the efficacy and biocompatibility of a wide variety of compounds [43]. In the present study, the in ovo method was used to evaluate the biocompatibility of Methotrexate and Cetuximab on the vascular plexus. The data obtained indicated that both compounds have an average irritation score, Methotrexate producing more irritating effects than Cetuximab. In clinical practice, Methotrexate is used in doses between 12 and 500 mg/m^2^. Doses higher than 500 mg/m^2^ are sometimes used to treat cancers, such as lymphomas and osteosarcoma [44]. These doses can lead to toxic events, which sometimes increase the risk of morbidity and mortality and can lead to discontinuation of chemotherapy and cancer recurrence [44]. Towards its topical application, studies have suggested that systemic absorption is low, with toxic effects being much rarer than with oral or parenteral administration [45]. Wohlrab et al. [46] conducted a study on the biosafety of Methotrexate using the HET-CAM method, the results suggesting that when it was used in concentrations of 0.25% and 0.5%, it does not cause significant changes in the vascular plexus. These data are correlated with the results obtained, which shows that Methotrexate has a medium risk of irritation when used at a concentration of 150 µg/mL.

The maximum tolerated dose of Cet is not established, the optimal dose being established according to receptor saturation. However, when used in therapeutic doses, the main toxic effects associated with Cet treatment are skin reactions [47]. Regarding the effects recorded in this study on the chorioallatoic membrane, Cetuximab showed a weak irritating effect, with an irritation score of 1.75. At the endothelial level, there are numerous markers involved in the production of vascular pathologies, such as schizophrenia [48], asphyxia [49] but also the neoplastic process [50] and the irritating effect of the compounds [51]. The main agent involved in increasing vascular permeability and, implicitly, the irritating effect on the vascular plexus, is VEGF [52]. Following in vitro studies on the mechanism of action of Cetuximab, it has been shown that it has an anti-EGFR action, leading in parallel to downregulation of VEGF [53]. Decreased VEGF production may be one of the mechanisms underlying the biocompatibility of the compound in the chorioallatoic membrane. On the other hand, another marker characteristic of the irritating effect on vascular capillaries is IL-8. Elevated IL-8 levels are correlated with an irritating effect of the compounds tested on the chorioallatoic membrane [54]. IL-8 is also a key factor in EGFR signaling, being involved both in the production of toxic effects on the skin and in the stimulation of angiogenesis and metastasis [55]. Perotte et al. [56] analyzed the effect of Cetuximab on IL-8 expression, showing that Cetuximab acts by decreasing IL-8 protein production. Thus, by downregulating the production of factors involved in producing the irritating effect on the vascularization of the chorioallantoic membrane, Cetuximab has good biocompatibility, without major toxic effects. 

## 5. Conclusions

Met and Cet are two basic chemotherapies used successfully in cancer therapy. However, the toxic effects associated with them have not yet been fully elucidated. The results obtained in the current study showed that Met causes a dose-dependent cytotoxic effect in human keratinocytes. Cell death has been associated with up-regulation of pro-apoptotic markers (Bax and Bad). In addition, in the vascular plexus, Met caused irritant effects translated into hemorrhage, lysis, and vascular stasis. In comparison, Cet has a better safety profile, which does not lead to significant changes in cell viability, apoptotic gene expression, and vascular irritant effect. In conclusion, the use of Met raises toxicity issues, but nevertheless, additional in vivo studies are needed to fully elucidate the mechanisms leading to the toxic side-effects associated with the administration of these drugs.

## Figures and Tables

**Table 1:** Presentation of oligonucleotides corresponding to the primers used.

Primer	Forward	Reverse
18 S	5′ GTAACCCGTTGAACCCCATT 3′	5′ CCA-TCC-AAT-CGG-TAGTAG-CG 3′
Bcl-xL	5′GATCCCCATGGCAGCAGTAAAGCAAG 3′	5′CCCCATCCCGGAAGAGTTCATTCACT 3′
Bcl-2	5′ CGGGAGATGTCGCCCCTGGT 3′	5′ GCATGCTGGGGCCGTACAGT 3′
Bad	5′ CCC-AGA-GTT-TGA-GCC-GAG-TG 30	5′ CCC-ATC-CCT-TCG-TCC-T 3′
Bax	5′ GCCGGGTTGTCGCCCTTTT 3′	5′CCGCTCCCGGAGGAAGTCCA 3′

**Table 2:** Irritation score values for positive control (SDS 1%), negative control (distilled water), Met 150 µg/mL, and Cet 150 µg/mL.

	SDS 1%	H_2_O	Met 150 µg/mL	Cet 150 µg/mL
IS	19.41	0.14	7.83	1.75
tH	19 s	300	135	300
tL	23 s	297	196	285
tC	27 s	300	214	220
